# Sub-bandgap Photocurrent Spectra of p–i–n Perovskite Solar Cells with n-Doped Fullerene Electron Transport Layers and Bias Illumination

**DOI:** 10.1021/acsaem.4c01077

**Published:** 2024-07-11

**Authors:** Bas T. van Gorkom, Aron Simons, Willemijn H. M. Remmerswaal, Martijn M. Wienk, René A. J. Janssen

**Affiliations:** † Molecular Materials and Nanosystems & Institute for Complex Molecular Systems, Eindhoven University of Technology, P.O. Box 513, Eindhoven 5600 MB, Netherlands; ‡ Dutch Institute for Fundamental Energy Research, De Zaale 20, Eindhoven 5612 AJ, Netherlands

**Keywords:** perovskite solar cells, photocurrent spectroscopy, quasi-Fermi level splitting, sub-bandgap, defects, electron trap states

## Abstract

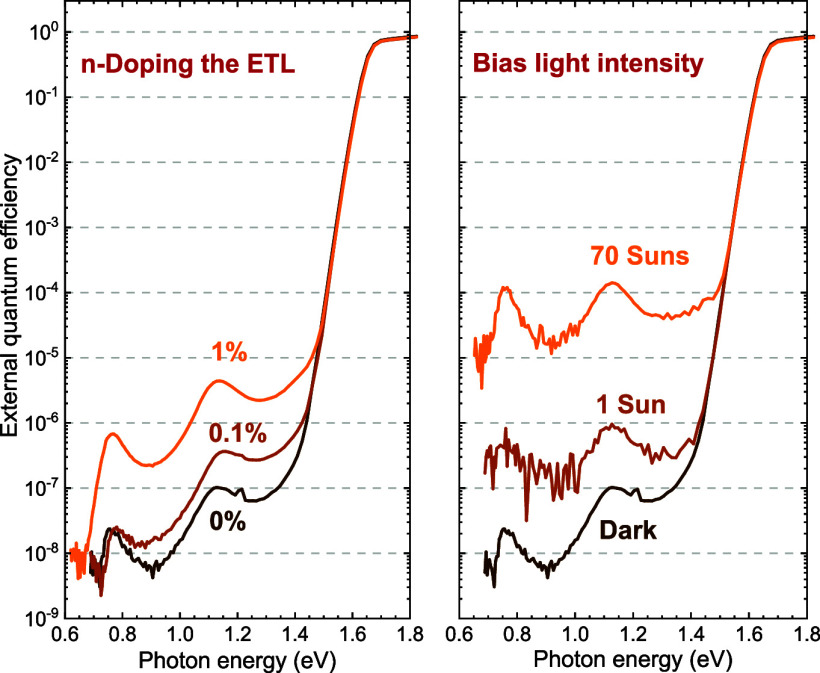

In p–i–n perovskite solar cells optical excitation of defect states at the interface between the perovskite and fullerene electron transport layer (ETL) creates a photocurrent responsible for a distinct sub-bandgap external quantum efficiency (EQE). The precise nature of these signals and their impact on cell performance are largely unknown. Here, the effect of n-doping the fullerene on the EQE spectra is studied. The n-doped fullerene is either deposited from solution or by coevaporation. The latter method is used to create undoped–doped fullerene bilayers and investigate the effect of the proximity of the doped region on the EQE spectra. The intensity of the sub-bandgap EQE increases when the ETL is n-doped and also when the device is biased with green light. Using these results, the sub-bandgap EQE signal is attributed to originate from electron trap states in the perovskite with an energy below the conduction band that are filled by excitation with low-energy photons. The trapped electrons give rise to photocurrent when they are collected at a nearby electrode. The enhanced sub-bandgap EQE observed when the ETL is n-doped or bias light is applied, is related to a higher probability to extract trapped electrons under these conditions.

## Introduction

The losses that occur in perovskite solar cells (PSCs) compared to the radiative detailed-balance limit mainly find their origin in nonradiative recombination of photogenerated charges. Especially for wide-bandgap and to a lesser extent for narrow- and intermediate-bandgap PSCs the deficit between the open-circuit voltage energy and bandgap must be reduced to further increase efficiencies. Nonradiative recombination occurs in the bulk,^1^ at grain boundaries,^2^ or at interfaces,^3^ and is often mediated by defects such as vacancies and interstitials. Recent PSC research has focused on strategies to reduce or passivate defects.^4^ While perovskite defect chemistry has been studied in detail theoretically,^5^ experimental evidence of the nature of the defects, their location, and even their energy is more difficult to obtain, despite a plethora of experimental techniques to investigate their nature.^6^ To address this issue and to understand the precise cause of passivation via optimized solution processing, thermal annealing, or bulk and interfacial additives that have been developed in recent years, it is necessary to investigate and understand perovskite defects in more detail.

Photocurrent spectroscopy has been used to study defects in perovskite semiconductors.^7−12^ It is a highly sensitive technique with a detection range of up to 10 orders of magnitude in terms of the external quantum efficiency (EQE). Extracting the energy of the defects is, however, complicated by the fact that the spectral characteristics in thin film solar cells are strongly susceptible to interference of light.^13,14^ Previously we used photocurrent spectroscopy to probe defect states in PSCs^15^ and perovskite photodiodes.^16^ For p–i–n solar cells, with a perovskite layer sandwiched between a polytriarylamine (PTAA) hole selective contact and a [6,6]-phenyl-C_61_-butyric acid methyl ester (PCBM) electron selective contact, two sub-bandgap defects were identified. By using two transparent contacts embedded in a device configuration with a magnesium fluoride (MgF_2_) optical spacer and a silver (Ag) reflector, sub-bandgap signals at photon energies of 0.72 and 1.09 eV were identified that originate from defects positioned close to the perovskite–PCBM interface.^15^ However, the role of the PCBM layer and the precise nature of these sub-bandgap signals and associated defects remains elusive.

To further elucidate the role of PCBM, this study focuses on p–i–n PSCs in which the PCBM layer is n-doped to investigate the relation between the measured sensitive EQE and doping of the PCBM electron transport layer (ETL). While doping improves the electrical conductivity of organic semiconductors, it has also been associated with increased interfacial recombination in PSCs.^17^ Free charges created by doping of the charge selective contacts can potentially promote recombination of charge carriers generated in the perovskite absorber layer.^3^ In addition to homogeneously n-doped PCBM films, we study vapor-deposited Buckminsterfullerene (C_60_) films, n-doped by coevaporation of a dopant, to fabricate defined bilayers of undoped and doped C_60_ to specifically assess the impact of doping at the interface with the perovskite film. Finally, the effects of continuous illumination, i.e., bias light with energies above and below the bandgap, on the EQE is investigated. The results provide evidence that PCBM and C_60_ are facilitating the extraction of electrons, but are not the origin of the defects. Instead the defects originate from the perovskite semiconductor.

## Experimental Section

### Solution Preparation

PTAA (*M*_w_ = 14.5 kg mol^–1^, EM Index Co. Ltd.) (3 mg) was dissolved in toluene (Sigma-Aldrich, anhydrous) (1 mL) and stirred overnight at 60 °C in a nitrogen filled glovebox. 2-(9*H*-carbazol-9-yl)ethyl)phosphonic acid (2PACz) (98%, TCI) (0.3 mg) was dissolved in ethanol (Merck Milipore, anhydrous) (1 mL). PCBM (99%, Solenne BV) (20 mg) was dissolved in a 1:1 (v/v) mixture of chlorobenzene (CB) (Sigma-Aldrich, anhydrous) and chloroform (CF) (Sigma-Aldrich, anhydrous) (1 mL) and stirred overnight under ambient conditions. PCBM was doped with 4-(2,3-dihydro-1,3-dimethyl-1*H*-benzimidazol-2-yl)-*N*,*N*-dimethylbenzenamine (N-DMBI) (<98%, Sigma-Aldrich), which was dissolved in 1:1 (v/v) mixture of CB and CF. For the perovskite, two 1.5 M solutions containing lead iodide (PbI_2_, 99.99%, trace metal basis, TCI Chemicals) and lead bromide (PbBr_2_, >98%, TCI Chemicals) were prepared in a 4:1 (v/v) mixture of *N,N*-dimethylformamide (DMF, Sigma-Aldrich, anhydrous, 99.8%) and dimethyl sulfoxide (DMSO, Sigma-Aldrich, anhydrous, 99.9%) and stirred overnight at 60 °C inside a nitrogen filled glovebox. PbI_2_ (936 μL at 1.5 M) was mixed with formamidinium iodide (FAI, Greatcell Solar) (200 mg) and PbBr_2_ (936 μL at 1.5 M) with methylammonium bromide (MABr, Greatcell Solar) (133 mg) and stirred at 60 °C for ∼1 h. The resulting FAPbI_3_ and MAPbBr_3_ solutions were mixed in a volumetric ratio of 4.6:1. Additionally, 5 vol % of cesium iodide (CsI, anhydrous, beads, 99.999%, Sigma-Aldrich) dissolved (1.5 M) in DMSO was added and the resulting solution was stirred at 60 °C for ∼1 h inside a nitrogen filled glovebox.

### Device Fabrication

Patterned indium tin oxide (ITO) substrates (Naranjo Substrates) were cleaned by sonication in acetone; scrubbing and sonication in a solution of sodium dodecyl sulfate (SDS, Acros, 99%) in water; rinsed with deionized water; and sonicated in 2-propanol. The substrates were dried and treated in a UV-ozone oven for 30 min, shortly before use. The substrates were transferred to a nitrogen-filled glovebox for spin coating of the charge transport and perovskite layers. The PTAA solution was spin coated at 5800 rpm for 30 s, followed by annealing at 100 °C for 10 min. The 2PACz solution was spin coated at 3000 rpm for 30 s, followed by annealing at 100 °C for 10 min. The substrate was allowed to cool to room temperature before the perovskite precursor solution was spin-coated onto the substrate at 4000 rpm (at an acceleration of 800 rpm s^–1^) for 40 s. After 25 s, 300 μL of antisolvent (ethyl acetate (Sigma-Aldrich, anhydrous, 99.8%) was dropped onto the spinning substrate. The sample was annealed at 100 °C for 30 min. The PCBM solution was spin coated at 1000 rpm for 60 s, followed by annealing at 100 °C for 30 min. C_60_, leucocrystal violet (LCV), bathocuproine (BCP), lithium fluoride (LiF), aluminum (Al) and Ag were all thermally evaporated onto the samples under high vacuum (∼3 × 10^–7^ mbar). The overlap between the ITO front and Al, or Ag, back contact (0.09 cm^2^ or 0.16 cm^2^) determined the active area of the solar cells.

### Device Characterization

Current density voltage (*J*–*V*) characteristics of the solar cells were measured with a Keithley 2400 source measure unit in a dry and oxygen free nitrogen atmosphere (<1 ppm of O_2_ and <1 ppm of H_2_O). A tungsten halogen lamp, filtered by a Schott GG385 UV filter and a Hoya LB120 daylight filter, was used to simulate the air mass 1.5 globally diffuse (AM1.5G) solar spectrum at 100 mW cm^–2^, calibrated by a silicon (Si) photodiode. No preconditioning of the cells was used before characterization. A shadow mask of 0.0676 cm^2^ was used to define the cell area. *J*–*V* scans involved sweeping the applied voltage (with no prebiasing) from +1.5 to −0.5 V for a reverse scan or from −0.5 to +1.5 V for a forward scan at a rate of 0.25 V s^–1^. To measure EQE, the cells were contacted in a nitrogen-filled container. A 50 W tungsten halogen lamp was used as light source. The light was chopped at 158 Hz before passing into a monochromator (Oriel, Cornerstone 130). A reference silicon detector was used to calibrate the current from the cell which was fed into a current preamplifier (Stanford Research, SR 570). The resulting voltage was measured using a lock-in amplifier (Stanford Research, SR 830). A green light-emitting diode (LED) (Thorlabs, M530L3) was used as a light bias to generate approximately 1-Sun equivalent illumination intensity. Integration of the EQE with the AM1.5G spectrum afforded estimates of short-circuit current density (*J*_sc_) that were within 4% of the values measured with the simulated solar light.

### Sensitive Photocurrent Spectroscopy

To measure sensitive EQE spectra, an Oriel 3502 light chopper, Cornerstone 260 monochromator (CS260-USB-3-MC-A), a Stanford Research SR 570 low-noise current preamplifier, a Stanford Research SR830 lock-in amplifier, and a 250 W tungsten-halogen lamp were used. The light was chopped at a frequency of 330 Hz. A series of long pass filters (optical density, OD ≥ 5) with increasing cut-on wavelengths was placed between the lamp and monochromator to remove stray light during the measurement. The monochromatic light is then passed through a concave cylindrical lens, to focus the light and increase the intensity on the active area of the solar cell. The solar cell was kept in an electrically insulated nitrogen-filled container. The cell is contacted using spring-loaded gold contacts. The current generated by the solar cell is fed into the preamplifier via a triaxial cable which is kept at a distance from other cabling to minimize the spurious signals due to induction. Above the bandgap a sensitivity of 200 μA V^–1^ was used for the preamplifier and this was increased to 200 nA V^–1^ to measure signals below the bandgap. The lock-in amplifier was set in float mode to reduce background noise and a time constant of 1 s and a settling time of 15 s were used. Calibrated Si and indium gallium arsenide (InGaAs) photodiodes were used to determine incident light intensity. Two lasers (532 nm, 30 mW, B&W Tek Inc. and 1319 nm, 200 mW, Changchun New Industries Optoelectronics Tech. Co.) were used as bias sources.

### Quasi-Fermi Level Splitting

The sample was illuminated using a 455 nm Thorlabs M455F3 LED. The light passed through an in-fiber filter holder containing an Edmund Optics 550 nm short-pass filter before entering an integrating sphere (Avantes AvaSphere-30-REFL). The photoluminescence (PL) emission from the sample was collected through an optical fiber mounted to the side of the sphere and passed via an in-fiber filter holder with an Edmund Optics 550 nm long-pass filter to a spectrometer (Avantes AvaSpec-HSC1024X58TEC-EVO). The setup is calibrated using an Avantes halogen lamp with known spectral irradiance. Measurements were performed at 1-Sun equivalent conditions.

## Results and Discussion

### Sensitive EQE with Bulk Doped ETLs

N-DMBI was used as dopant for PCBM. Although the exact mechanism and molecular species involved remain somewhat unclear, the doping mechanism involves a combination of a hydride transfer and an electron transfer.^18−21^ Layers of PCBM containing different amounts of N-DMBI were spin-coated from a 1:1 v/v mixture of chloroform and chlorobenzene and annealed at 100 °C for 30 min under inert conditions. To confirm the n-doping, two Al contacts spaced 2 mm apart were fabricated on top of the film using thermal evaporation. The current–voltage (*I*–*V*) characteristics of PCBM films doped show increased conductivity upon addition of 0.1 wt % of N-DMBI (Figure S1). The conductivity decreases slightly for 1 wt %. This is attributed to the poor solubility of the PCBM^•–^ and N-DMBI^+^ radical ions in the solvent mixture used to process the film. The radical ions readily precipitate resulting in a poor film morphology and impeded (lateral) charge transport.^22,23^ Solution processing of uniform n-doped PCBM films is challenging because precipitation occurs within minutes after preparation of the doped solution. Solvents that might provide a higher solubility for the ions exhibit a lower solubility for PCBM and do not improve the tedious layer deposition.

**Figure 1:**
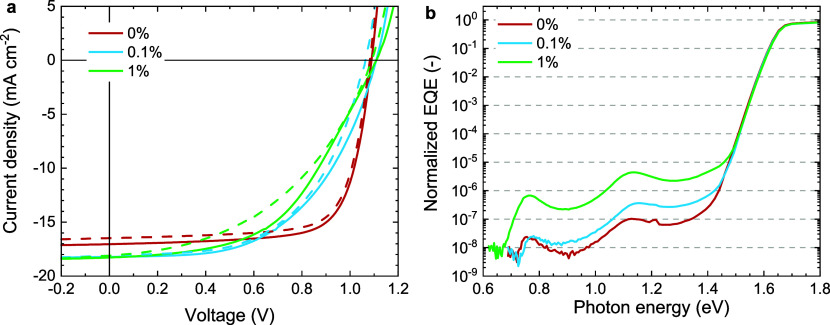
Characterization of glass|ITO|PTAA|Cs_0.05_(FA_0.83_MA_0.17_)_0.95_Pb(I_0.83_Br_0.17_)_3_|ETL|LiF|Al p–i–n PSCs, using PCBM with different amounts of N-DMBI as ETL. The amounts of N-DMBI used to dope the PCBM is shown in wt % in the legend. (a) *J*–*V* characteristics in reverse (solid lines) and forward (dashed lines) scan directions recorded under simulated AM1.5G illumination at 100 mW cm^–2^ as described in te Experimental Section. (b) Sensitive EQE spectra. The spectra were normalized to 1 at 480 nm, corresponding to the maximum EQE in each spectrum.

To study the influence of n-doping on solar cell performance, three p–i–n perovskite cells were fabricated using PTAA as hole transport layer and PCBM with 0, 0.1, and 1 wt % N-DMBI as ETL. The nominal perovskite composition is Cs_0.05_(FA_0.83_MA_0.17_)_0.95_Pb(I_0.83_Br_0.17_)_3_ (FA is formamidinium, MA is methylammonium) with a bandgap of 1.64 eV and the deposition is based on an antisolvent recipe.^24^ The front electrode was ITO, while LiF (1 nm) with Al (100 nm) were used as back electrode. For 0.1 and 1 wt % N-DMBI a small increase in *J*_sc_ is observed compared to the reference device (Figure 1a and Table S1). However, this is accompanied by a decrease in fill factor (FF). At higher concentrations (5 wt % N-DMBI) the device performance dropped to low levels as a result of the solubility issues of the PCBM/N-DMBI complex,^25^ and the associated poor morphology of the n-doped PCBM films.

Figure 1b shows that the EQE intensity in the sub-bandgap region increases when the PCBM layer is n-doped. The spectra were normalized to unity at 480 nm where they reached the maximum value. For 1 wt % of N-DMBI, the defect-related signal increases by one and a half order of magnitude, while the shape of the signal remains the same. The distinct peaks at 0.76 and 1.12 eV result from optical interference effects.^12−15^

**Figure 2:**
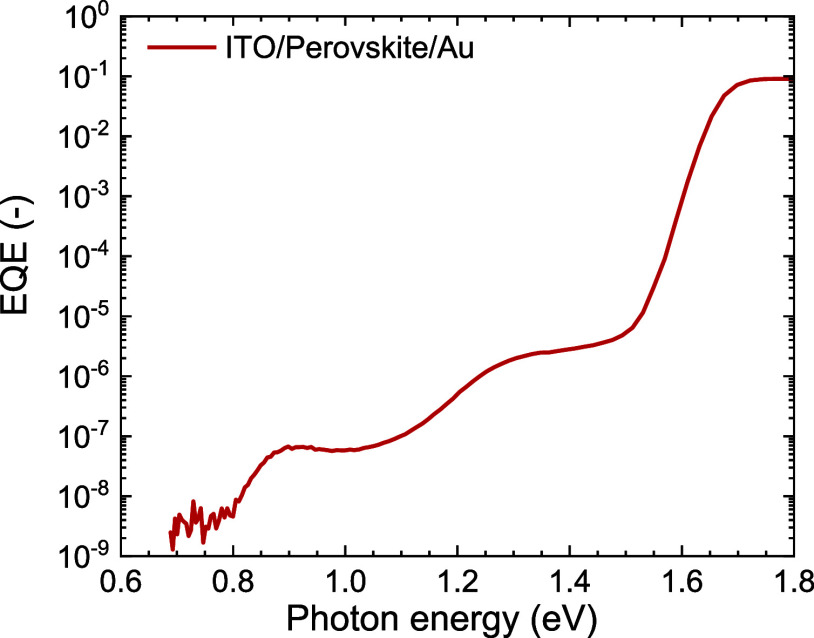
Sensitive EQE spectrum of an ITO|Cs_0.05_(FA_0.75_MA_0.25_)_0.95_Pb(I_0.83_Br_0.17_)_3_|Au device. The spectrum is not normalized and was measured directly after fabrication to minimize effects of Au migration.^29^

To rule out a scenario where the sub-bandgap EQE is solely a result of the PCBM contributing to the photocurrent, a PSC was fabricated without charge selective layers. For this experiment, the perovskite layer was sandwiched between an ITO bottom electrode and a gold (Au) top electrode. Perovskite–metal interfaces are rather unstable. Metals like aluminum,^26^ copper,^27^ and silver^28^ react with the halides of the perovskite. Au was chosen as contact here because it is more inert compared to the other metals. Au is known to migrate through the perovskite film,^29^ providing a potentially detrimental effect on the EQE measurement. To minimize this effect, the devices were measured directly after fabrication. Interestingly, without using charge selective contacts, the same two features appear in the sub-bandgap EQE as for the devices made with PTAA and PCBM layers (Figure 2). The slight blue shift in peak positions in Figure 2 (about 0.9 and 1.3 eV) compared to Figure 1b (0.76 and 1.12 eV) is ascribed to the difference in optical interference in the layer stacks due the absence of charge transport layers in the devise that only uses ITO and Au as electrodes.^15^ The large similarity between the spectra demonstrates that the sub-bandgap EQE signals likely originate from the perovskite film and are not caused by PCBM. Alternatively, the sub-bandgap EQE signal can result from interfacial defects formed at perovskite–fullerene or perovskite–metal interfaces.^30^ The increased sub-bandgap EQE introduced by n-doping of PCBM is therefore either due to filling of (existing or formed) interfacial traps, or because n-doping facilitates the extraction of trapped charge carriers at the perovskite–PCBM interface.

To explain the fact that the sub-bandgap EQE signals results from electron hole-pairs generated near the perovskite–fullerene interface,^15^ but are not caused by the fullerene, we consider as a tentative explanation that the pristine perovskite film has electron trap states below the conduction band that can be filled by sub-bandgap optical excitation from the valence band. In the bulk, these electrons are trapped and consequently, they will recombine with (geminate) holes and not produce a photocurrent. We conjecture that only when created close to the interface with an ETL or metal contact, the trapped electrons in the perovskite have a finite probability to be collected, after which the associated holes can be collected at the opposite electrode to produce a net photocurrent. The effect of the increased sub-bandgap EQE when using an n-doped PCBM layer can then be rationalized in two ways. First, free electrons from the n-doped PCBM can partially fill electron trap states in the perovskite near the interface with PCBM and improve the probability that electrons excited by sub-bandgap photons at distances further from the interface can be collected because of improved transport.^31^ A second explanation is that the ability of PCBM to extract trapped charge carriers is enhanced when it is n-doped. It is known that the lowest unoccupied molecular orbital (LUMO) of PCBM is lowered upon doping, which increases the driving force for extraction of trapped electrons.^31^ Vibronic states existing below the LUMO of PCBM have also been suggested as being the cause of defect-related signals and might also play a role here.^32^ Regardless of the exact nature of the states, the ability of PCBM to extract trapped electrons determines the sub-bandgap EQE intensity and n-doping enhances this ability.

### Sensitive EQE with Undoped–Doped Bilayer-ETLs

Having established the strong dependence of the sub-bandgap EQE on the doping level of PCBM and the dominant role in general of the interface between the perovskite and PCBM, better control over the spatial distribution of the doping relative to this interface can help to shed light on the mechanism that produces sub-bandgap EQE signals. The solution doping employed for PCBM results in bulk n-doping. A bilayer of undoped and doped C_60_ can be fabricated by thermal evaporation of C_60_, followed by a coevaporation of C_60_ with a dopant molecule. By adapting the thickness of the first layer, the spatial distance between the n-doped C_60_ and the perovskite can be controlled. Combinations of an undoped and doped layer are commonly used in the field of organic light-emitting diodes (OLEDs)^33^ and have also been used in PSCs.^34,35^

Instead of N-DMBI, LCV is used as n-dopant for C_60_. LCV can be thermally evaporated, allowing for a coevaporation process with C_60_ as ETL. LCV has been reported to n-dope C_60_ after thermal or UV treatment.^36^ The deposition rates of C_60_ and LCV were 0.5 and 0.05 Å s^–1^, respectively. This resulted in a final film of doped C_60_ with around 7 wt % of LCV. The doping mechanism has been proposed to proceed via an electron transfer from an excited LCV molecule to a C_60_ molecule.^36^ This electron transfer step would require thermal or photo excitation and results in the formation of a fullerene radical anion, C_60_^•–^, and an LCV radical cation, LCV^•+^. The back electron transfer is inhibited when LCV^•+^ loses a hydrogen atom (H^•^) in an irreversible step forming the closed-shell crystal violet cation, CV^+^. To test the doping, films were fabricated by coevaporation of 50 nm C_60_ with 5 nm LCV. The conductivity was measured with a set of interdigitated electrodes spaced 0.2 mm apart. In addition to coevaporated films of C_60_ and LCV, three films were fabricated where 1, 3, or 5 nm of LCV was deposited sequentially on top of 50 nm of the C_60_ layer to see whether doping could occur by diffusion of the LCV into the C_60_ film. For all films, an increase in conductivity is observed compared to pristine C_60_ (Figure S2), but it is significantly less than for PCBM doped with lower amounts of N-DMBI (Figure S1). This indicates that doping by LCV is less efficient and most LCV will remain in its pristine, unreacted form. The highest lateral conductivity is observed after evaporating LCV (5 nm) on top of a pristine C_60_ layer. In that case the doping level of C_60_ at the top surface can be expected to be high. Also for the coevaporated LCV where bulk doping of C_60_ occurs, a distinct increase in conductivity is measured. For these films, no further increase in either conductivity was measured upon annealing or UV soaking. Apparently, n-doping occurs during the evaporation process itself, possibly by in situ activation of the LCV molecules.

**Figure 3:**
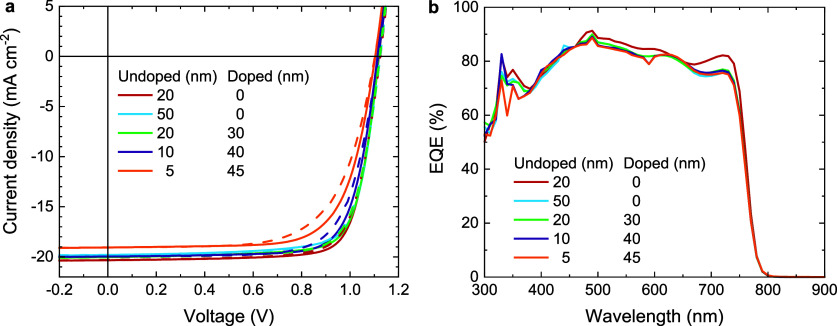
*J*–*V* characteristics of glass|ITO|2PACz|Cs_0.05_(FA_0.83_MA_0.17_)_0.95_Pb(I_0.83_Br_0.17_)_3_|ETL|BCP|Ag solar cells. The ETL consists of either undoped C_60_ of 20 or 50 nm, or a combination of an undoped C_60_ of 20, 10, and 5 nm followed by 30, 40, and 45 nm doped C_60_, respectively. The C_60_ is doped via coevaporation of LCV. (a) *J*–*V* characteristics with reverse and forward scan directions are shown in the solid and dashed lines, respectively. (b) Corresponding EQE spectra.

Glass|ITO|2PACz|Cs_0.05_(FA_0.75_MA_0.25_)_0.95_Pb(I_0.83_Br_0.17_)_3_|ETL|BCP|Ag solar cells were fabricated using a self-assembled monolayer of 2PACz on ITO as front electrode and different thicknesses of undoped and LCV-doped C_60_ with BCP and Ag as back electrode The thickness of C_60_ typically used for these devices is around 20 nm. To allow for a wider range of thickness variations of the doped films, the total layer of the pristine and doped C_60_ films was kept at 50 nm total, e.g., 5 nm pristine C_60_ followed by 45 nm of C_60_ doped with LCV. Undoped C_60_ was used at the interface with the perovskite film to minimize interaction of doped region with the perovskite film. The thicknesses of the undoped/doped C_60_ bilayers were 5/45, 10/40, 20/30 (nm/nm). A device using 50 nm of undoped C_60_ was fabricated to account for any thickness effects on device performance. The *J–V* characteristics (Figure 3a) and photovoltaic parameters of these devices (Table S2) reveal that n-doping of C_60_ in a bilayer configuration has a minimal impact on the device performance. Only when a bilayer of 5 nm undoped and 45 nm of doped C_60_ is used as ETL, a small decrease in open-circuit voltage (*V*_oc_), *J*_sc_, and FF is observed. This 5/45 bilayer corresponds to the highest doping, indicating that doping of C_60_ has no beneficial effects on device performance. Above the bandgap, the EQE spectra of the devices that have (combined) ETL thickness of 50 nm are virtually identical (Figure 3b). The EQE for the device with 20 nm of undoped C_60_ as ETL is slightly higher around 700 nm because of a change in the interference of light.

**Figure 4:**
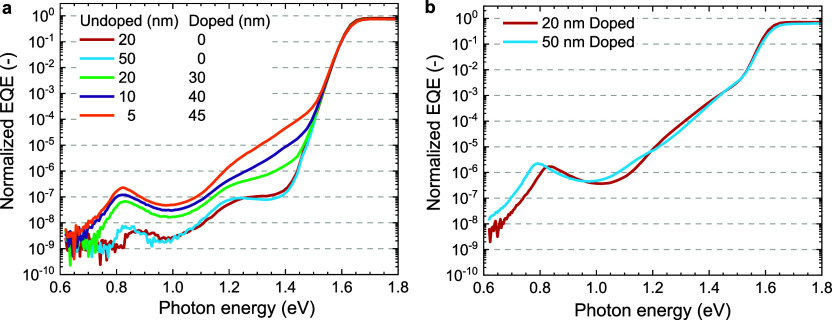
Sensitive EQE of glass|ITO|2PACz|Cs_0.05_(FA_0.83_MA_0.17_)_0.95_Pb(I_0.83_Br_0.17_)_3_|ETL|BCP|Ag solar cells. (a) For cells using either 20 or 50 nm pristine C_60_ or a combination of 20, 10, and 5 nm of pristine C_60_ with 30, 40, and 45 nm doped C_60_, respectively. (b) For cells using 20 or 50 nm of doped C_60_. The spectra were normalized to 1 at 490 nm, corresponding to the maximum EQE in each spectrum.

The sensitive EQE spectra, normalized to 1 at 490 nm, of these devices (Figure 4) allow investigating the influence of the doping and the distance of the n-doped C_60_ layer to the perovskite interface on the sub-bandgap photocurrent. The sub-bandgap EQE of devices with 20 and 50 nm of undoped C_60_ as ETL have similar intensity. There is a slight shift of the peaks to lower photon energies for the device using 50 nm, which is a consequence of the increasing thickness of the stack which in turn changes the interference of the optical electric field as discussed in detail previoulsy.^15^ The devices with a bilayer of undoped and doped C_60_ show a distinct increase in sub-bandgap EQE. The defect-related signal at 0.8 eV increases by an order of magnitude when 20 nm pristine and 30 nm doped C_60_ are used. The signal increases further for the devices where the undoped C_60_ layer is thinner and the LCV-doped C_60_ layer becomes thicker. This indicates that the presence of doping near the perovskite interface is responsible for the increase in sub-bandgap EQE. One issue to consider is that the total amount of doped C_60_ decreases when the undoped layer becomes thicker. To rule out the influence of the total amount of doping, two devices (20 and 50 nm) were also fabricated in which C_60_ is doped with 7 wt % of LCV. The sub-bandgap EQE of these devices have very similar intensity, but for 50 nm the signals shift to lower energies due a change in optical interference (Figure 4b). This demonstrates that the signal does not depend on the total amount of doped C_60_ present. The sub-bandgap EQE signal for both devices (Figure 4b) are higher than for the bilayer devices (Figure 4a). This demonstrates that the increase in sub-bandgap EQE is related to the presence of n-doping of C_60_ at the perovskite–C_60_ interface.

A notable difference between the sensitive EQE spectra for solar cells with a doped PCBM layer (Figure 1b) and a doped-C_60_ layer (Figure 4a), is an additional signal at about 1.35 eV when LCV is used to dope C_60_. This signal is ascribed to the photoinduced charge-transfer (CT) transition between LCV and C_60_ (C_60_ + LCV + *h*ν → C_60_^•–^ + LCV^•+^).^37,38^ CT state formation is the primary step in photodoping, but reversible when not followed by the loss of hydrogen (LCV^•+^ → CV^+^ + H^•^). Because the doping efficiency of LCV is low,^36^ most LCV is in its pristine form and the CT excitation contributes to the EQE. This contribution becomes significantly more intense than the signal at 0.8 eV, when the thickness of the undoped layer decreases from 20, via 10, to 5 nm. The fact that a CT contribution is measured from a doped C_60_ layer on top of a pristine C_60_ layer, indicates that holes formed on LCV, can be transported through a pristine layer of C_60_ and be injected into the perovskite film, after which they are transported to the hole extracting contact.

Another issue to consider is that while the EQE in the sub-bandgap region increases by over an order of magnitude when the C_60_ is doped, the device performance remains almost unaffected (Figure 3 and Table 1). To investigate whether n-doping introduces additional nonradiative recombination losses, the quasi-Fermi level splitting (QFLS) of perovskite films covered with the same ETLs as used in the solar cells shown in Figure 3a was determined by measuring the absolute photoluminescence photon flux under 1-Sun equivalent illumination (Figure S3). Table 1 shows that the level of doping used does not significantly affect the QFLS and, hence, the nonradiative recombination does not change significantly, consistent with the nearly identical *V*_oc_ of the solar cells (Figure 3a). Differences in QFLS between illumination from the glass or C_60_ side are uniform among the samples and attributed to parasitic absorption by the C_60_ layer and differences in light in- and outcoupling. The relation between the intensity of the sub-bandgap EQE and nonradiative recombination is not direct, because the EQE in the sub-bandgap region increases significantly with n-doping while the *V*_oc_ and QFLS are constant.^32^

**Table 1:** QFLS of Glass|Cs_0.05_(FA_0.75_MA_0.25_)_0.95_Pb(I_0.83_Br_0.17_)_3_|C_60_ Layers for Different Undoped/Doped C_60_ Bilayers Determined for Illumination from the Glass or C_60_ Side

*d*_undoped_ (nm)	*d*_doped_ (nm)	illumination side	QFLS (meV)^a^
20	0	glass	1095 ± 11
		C_60_	1071 ± 17
50	0	glass	1102 ± 11
		C_60_	1074 ± 14
20	30	glass	1108 ± 12
		C_60_	1085 ± 11
10	40	glass	1100 ± 12
		C_60_	1065 ± 12
5	45	glass	1106 ± 11
		C_60_	1088 ± 12

a Standard deviations are based on 4 measurements.

The experiments with undoped/n-doped C_60_ bilayers confirm that n-doping enhances the defect-related sub-bandgap photocurrent and EQE and does so more significantly when the doped region is closer to the perovskite layer. The experiments further show that the nonradiative recombination is not affected at the doping levels used. These experiments support the idea that n-doping of the fullerene ETL at the doping levels used, enhances the extraction of trapped electrons created by sub-bandgap optical excitation under short-circuit conditions but does not significantly affect nonradiative recombination at open circuit.

### Effect of Light Intensity on the Sensitive EQE

**Figure 5:**
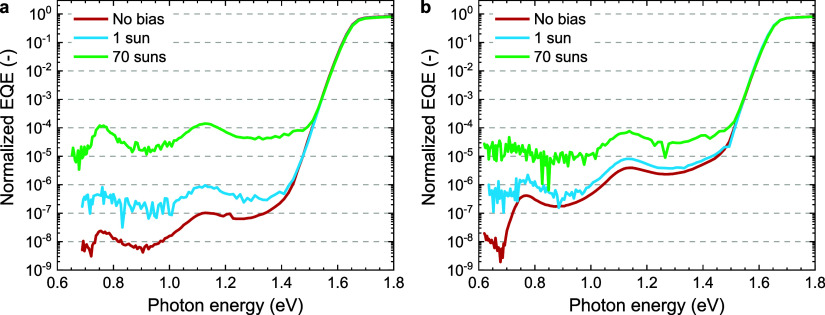
Sensitive EQE of glass|ITO|PTAA|Cs_0.05_(FA_0.83_MA_0.18_)_0.95_Pb(I_0.83_Br_0.17_)_3_|ETL|LiF|Al solar cells, biased with a 532 nm laser at 1 and 70 Suns intensity. (a) Using undoped PCBM as ETL. (b) Using PCBM doped with 1 wt % N-DMBI as ETL.

When measuring sensitive EQE spectra the intensity (power) of the mechanically chopped probe light is maximized to improve the signal-to-noise ratio. To evaluate the impact of additional charge carriers, sensitive EQE measurements with continuous bias illumination were performed. Continuous bias illumination generates a constant photocurrent that is not registered by the lock-in amplifier to detect the EQE but is amplified by the preamplifier before being filtered out. This limits the gain that can be applied and ultimately the dynamic range of the experiment. The increase in current also raises the noise floor of the setup.^39^ A continuous-wave (CW) laser (532 nm) was used for light-biassing and a plano-convex lens was placed between the laser beam and the cell to ensure that the full cell area is illuminated. A series of neutral density filters was used to adjust the intensity of the bias light. The cells were biased with an intensity that resulted in a *J*_sc_ similar to the value measured under simulated AM1.5G and 100 mW cm^–2^ irradiance (1-Sun equivalent). In a second experiment, the intensity was increased to around 70 Suns. With 1 Sun bias illumination the EQE signals at 0.75 and 1.15 eV for the cell with an undoped PCBM ETL (Figure 5a) increase by factors of about 20 and 10, respectively. When the light bias is increased to 70 Suns, the same signals increase by factors of 5000 and 1000. For the doped PCBM sample (Figure 5b), the signals at 0.75 and 1.15 eV increase by a factors of 4 and 2 under 1 Sun bias, and by factors of 40 and 16 at 70 Sun bias. Clearly, the sub-bandgap EQE signals strongly dependent on the presence of additional charge carriers. For undoped PCBM, the increase in EQE with light bias is initially more than for the n-doped PCBM ETL, but under 70-Sun bias the EQE signals for the pristine PCBM ETL and n-doped sample become similar. Overall, these results suggest that n-doping and light biasing fulfill a phenomenologically similar role in increasing the sub-bandgap EQE and we explain the possible cause in the next section.

**Figure 6:**
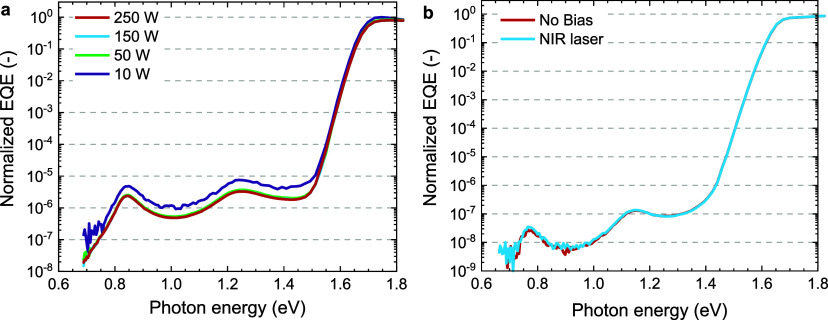
Sub-bandgap EQE of glass||ITO| PTAA|Cs_0.05_(FA_0.83_MA_0.17_)_0.95_Pb(I_0.83_Br_0.17_)_3_|ETL|LiF|Al. (a) Using PCBM doped with 0.5 wt % N-DMBI as ETL where the power of the tungsten halogen lamp which is used as probe light is decreased from 250 W (reference) to 150, 50, and 10 W. (b) Using pristine PCBM as ETL measured without any bias or biased with a 1319 nm laser.

Another possible explanation for sub-bandgap photocurrent is a two-photon process, which has been observed for organic semiconductors^40^ and some perovskites.^41,42^ In two-photon absorption, two photons with energy less than the bandgap are simultaneously absorbed and create an electron hole pair. Two-photon absorption usually requires high light intensities because the absorption cross sections are low,^41,42^ but when it proceeds via a defect state at midbandgap energies it can also occur at lower light intensities.^40^ To investigate whether a two-photon process is involved in generating sub-bandgap photocurrent, the intensity of the probe light was varied. The power of the tungsten halogen lamp used in these experiments was adjusted from 250, via 150, and 50, to 10 W, resulting in an attenuation of light intensity by factors of about 1.7, 7.7, and 700, respectively and the sub-bandgap EQE was measured at these four intensities for a cell with an n-doped PCBM ETL. If the sub-bandgap photocurrent would rely on a two-photon absorption process, a significant decrease is expected,^43^ but the sub-bandgap EQE was found to be virtually independent of the intensity of probe light (Figure 6a). Only for 10 W a small increase is observed. At this lowest light intensity noise in the photocurrent spectrum becomes more pronounced.

In addition to changing the probe light intensity, a p–i–n perovskite cell using pristine PCBM was biased with a 1319 nm laser (Figure 6b). If a two-photon process is involved, these photons should be able to either fill or empty the defect state at 0.8 eV. However, the NIR bias light does not significantly change the sub-bandgap EQE (Figure 6b). The independence of the sub-bandgap EQE on the intensity of probe light and the application of light bias with sub-bandgap wavelength rules out a two-photon absorption process as origin for the generation of sub-bandgap photocurrent in perovskites.

### Origin of the Sub-bandgap Photocurrent Signals

We previously demonstrated that the sub-bandgap EQE signals in p–i–n PSCs result from defect states that are located close to the interface between the perovskite and PCBM ETL,^15^ but we were unable to identify their nature in more detail. The experiments described in the previous sections give a clear answer to several outstanding questions regarding their origin. The sub-bandgap signals are not due to the fullerene ETL and therefore must be intrinsic to the perovskite or the perovskite–fullerene interface. The signals are also not caused by two-photon absorption in the perovskite. The sub-bandgap signals increase when using a n-doped fullerene layer that is near the perovskite interface and also increase when using green bias illumination to bring the solar cell close to, or well above, normal operation conditions. When doped with LCV, a contribution of an intermolecular photoinduced CT transition between LCV and C_60_ to the sub-bandgap EQE is seen. This implies that holes can be injected into the perovskite film and can be transported and extracted at the opposite electrode even at low concentration without becoming trapped or recombining.

**Figure 7:**
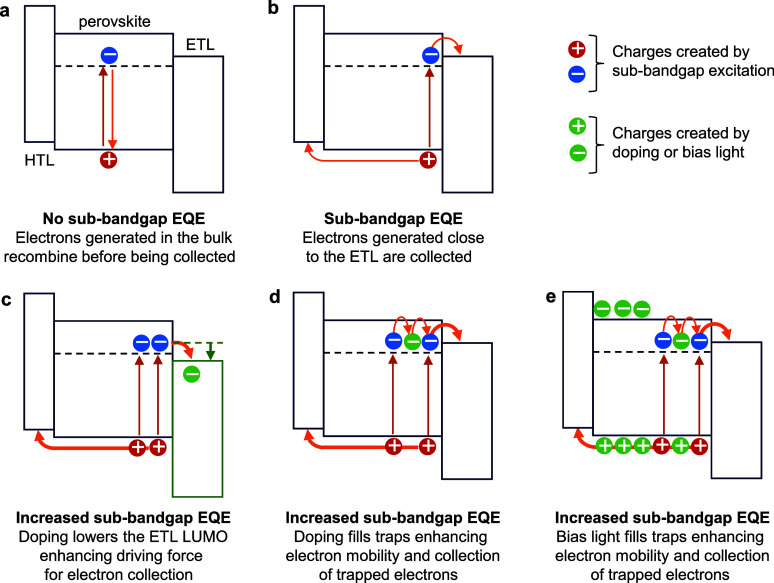
Schematic representation of the energy levels in p–i–n PSCs and the role of doping and bias light in increasing the sub-bandgap EQE. (a) Sub-bandgap excitation creates trapped electrons that cannot be collected. (b) Sub-bandgap excitation creates trapped electrons that can be collected when close to the ETL. (c) n-Doping decreases the LUMO of the fullerene layer and reduces the barrier for extracting trapped electrons. (d) n-Doping of the fullerene layer partly fills electron trap states in the perovskite, enhancing electron mobility and collection. (e) Bias light creates a background charge density that partly fills electron trap states in the perovskite and enhances electron mobility and collection. Note that the mechanisms presented in panels (c) and (d) can also act jointly.

The intensity of the defect-state related sub-bandgap EQE depends on a combination of the defect state density and a proportionality constant that involves, among others, the absorption coefficient and charge collection efficiency.^7^ If we consider the latter and assume that a low density of electron traps exists in the perovskite, the experimental observations can be rationalized. Sub-bandgap excitation in the bulk creates a trapped electron that recombines with a hole before it can be collected (Figure 7a). Only when the trapped electron is generated close to the fullerene (PCBM or C_60_) ETL it has a finite chance to be collected and, because the hole is free to move, a photocurrent is created that originates from a region close to the perovskite–fullerene interface (Figure 7b). Collection of these trapped electrons might involve thermal excitation. To explain the increase in sub-bandgap EQE upon n-doping the fullerene, two mechanisms can be envisioned. The first is based on a calculation of the density of states of molecular n-doped C_60_ which shows that n-doping lowers the LUMO of the fullerene.^44^ The lowering of the fullerene LUMO enhances the driving force for collecting electrons from the perovskites into the fullerene (Figure 7c). Also an undoped C_60_ layer between the perovskite and the doped C_60_ layers will experience a higher electron density. This explains the gradual loss in EQE signal intensity when the undoped region becomes thicker (Figure 4a). Second, n-doping of the ETL increases the electron density in the perovskite close to the interface and fills some of the traps and increase the mobility of excess electrons, such that more electrons created by sub-bandgap excitation can be collected (Figure 7d). We note that the two possible explanations do not exclude each other and might in fact both contribute. The same two mechanisms can also explain the increase in sub-bandgap EQE under green bias illumination. Charge carriers generated by above-bandgap bias light partially fill electron trap states and increase the mobility of electrons such that electrons excited by sub-bandgap photons can be collected (Figure 7e). Alternatively, the high constant photocurrent created by bias light causes a steady-state doping concentration of the fullerene ETL that is large enough to lower the LUMO and enhance the driving force for collection. It is not easy to decide which of these two possible mechanisms prevails, but the large increase in sub-bandgap EQE under green bias illumination seems to favor a mechanism where the enhanced electron mobility is the prime cause.

## Conclusions

The mechanism of sub-bandgap photocurrent generation has been studied in p–i–n PSCs. From the results we infer that sub-bandgap EQE signals likely originate from defects intrinsically present in the perovskite layer, but only appear when the fullerene ETL is able to extract trapped electrons from these defects. The extraction of trapped electrons then enables the geminate holes to move to the opposite electrode and sustain a photocurrent from sub-bandgap excitation. When the fullerene ETL is doped, the intensity of the defect-related sub-bandgap EQE increases significantly. This enhanced EQE depends on the presence of n-doping near the perovskite–fullerene interface and is attributed to an increased collection efficiency of the trapped electrons generated in the perovskite via sub-bandgap absorption. This increase can be explained by a downshift of the LUMO of the n-doped fullerene film or by an enhanced electron mobility when the n-doped fullerene party fills electron traps in the perovskite near their interface. Bias illumination also causes an increased sub-bandgap EQE. In this case the enhanced electron density improves the electron mobility and, possibly, also reduces the fullerene LUMO.

Finally, we note that the intensity of the sub-bandgap EQE signal is not straightforwardly related to the photovoltaic performance or nonradiative recombination. Doping of the ETL increases the sub-bandgap EQE by 1 order of magnitude (Figure 4a) but the changes in the *J*–*V* characteristics are modest (Figure 3a) and the QFLS is not affected (Table 1). This shows the limitations of highly sensitive photocurrent spectroscopy as a technique to explain differences in device performance. Hence, careful consideration should be given when interpreting sub-bandgap EQE spectra.

## Supporting Information Available

Conductivity of fullerene layers vs doping and table with photovoltaic parameters (PDF)
