# Supplementary material for: Sub-bandgap Photocurrent Spectra of p–i–n Perovskite Solar Cells with n-Doped Fullerene Electron Transport Layers and Bias Illumination
Source: ACS Appl Energy Mater. 2024 Jul 11;7(14):5869–78. doi: 10.1021/acsaem.4c01077 (PMC11267499; doi:10.1021/acsaem.4c01077)
Supplement: Supplementary file 1 — ae4c01077_si_001.pdf [file ae4c01077_si_001.pdf]

Supporting Information

Sub-Bandgap Photocurrent Spectra of p-i-n Perovskite Solar Cells with n-Doped Fullerene Electron Transport Layers and Bias Illumination

Bas T. van Gorkom,[1] Aron Simons, [1] Willemijn H. M. Remmerswaal, [1] Martijn M. Wienk, [1] and René A. J. Janssen[1,2]*

[1] Molecular Materials and Nanosystems & Institute for Complex Molecular Systems, Eindhoven University of Technology, P.O. Box 513, 5600 MB Eindhoven, The Netherlands

[2] Dutch Institute for Fundamental Energy Research, De Zaale 20, 5612 AJ Eindhoven, The Netherlands

* E-mail: r.a.j.janssen@tue.nl

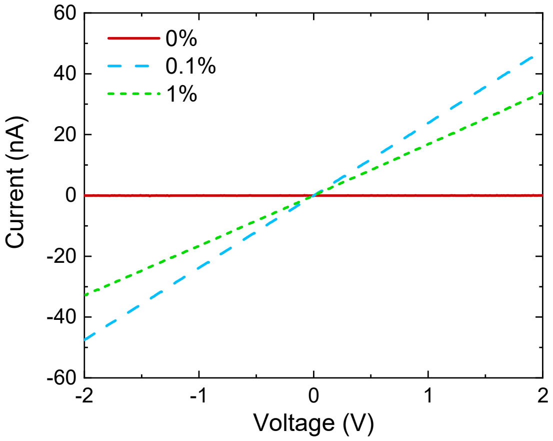

Figure S1. Current − voltage characteristics of PCBM films with 0, 0.1, and 1 wt.% N-DMBI spin-coated from of chloroform/chlorobenzene (1:1, v/v). The films were annealed at 100 °C for 30 min. Current was measured between Al bar-shaped electrodes at 2 mm spacing. Conductivities: $\sigma = 3.3 \times 10^{-3}$ mS cm^{-1} (0%), $\sigma = 2.4$ mS cm^{-1} (0.1%), $\sigma = 1.7$ mS cm^{-1} (1%).

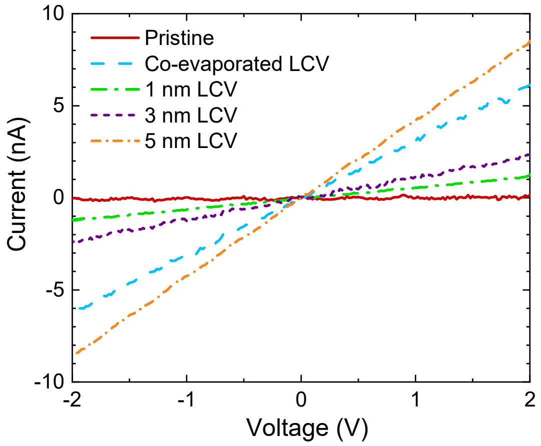

Figure S2. Current − voltage characteristics of 50 nm pristine C_{60}, 50 nm C_{60} with 5 nm co-evaporated LCV, and films of 50 nm C_{60} with 1, 3, and 5 nm LCV evaporated sequentially. Current was measured between Al bar-shaped electrodes at 0.2 mm spacing. Conductivities: $\sigma = 3.8 \times 10^{-3}$ μS cm^{-1} (pristine), $\sigma = 4.7$ μS cm^{-1} (Co-evap. LCV), $\sigma = 1.0$ μS cm^{-1} (1 nm LCV), $\sigma = 1.8$ μS cm^{-1} (2 nm LCV), $\sigma = 6.5$ μS cm^{-1} (5 nm LCV).

Table S1. Photovoltaic Parameters of Glass | ITO | PTAA | $Cs_{0.05}(FA_{0.83}\ MA_{0.17})_{0.95}Pb(I_{0.83}Br_{0.17})_3$ | ETL | LiF | Al Solar Cells Shown in Figure 1 of the Main Text.

Doping (%)	Scan	J_{sc} (mA cm^{-2})	V_{oc} (V)	FF	PCE (%)
0	Reverse	17.0	1.09	0.73	13.5
	Forward	16.5	1.08	0.74	13.1
0.1	Reverse	18.2	1.11	0.54	11.0
	Forward	18.1	1.06	0.57	10.9
1	Reverse	18.3	1.11	0.51	10.3
	Forward	18.1	1.09	0.46	9.0

Table S2. Photovoltaic Parameters of Glass | ITO | 2PACz | $Cs_{0.05}(FA_{0.83}MA_{0.17})_{0.95}Pb(I_{0.83}Br_{0.17})_3$ | ETL | BCP | Ag Solar Cells Shown in Figure 3 of the Main Text.

$d_{undoped}$ (nm)	d_{doped} (nm)	Scan	J_{sc} (mA cm^{-2})	J_{sc}^{EQE} (mA cm^{-2})	V_{oc} (V)	FF	PCE (%)
20	0	Reverse	20.3	20.6	1.12	0.77	17.8
		Forward	20.3		1.12	0.76	17.5
50	0	Reverse	19.8	19.8	1.12	0.76	16.9
		Forward	19.8		1.12	0.76	16.8
20	30	Reverse	20.0	20.0	1.12	0.77	17.2
		Forward	20.0		1.11	0.76	17.0
10	40	Reverse	20.0	19.9	1.11	0.76	16.7
		Forward	19.7		1.12	0.72	16.0
5	45	Reverse	19.1	19.8	1.10	0.71	15.4
		Forward	19.1		1.10	0.66	14.3

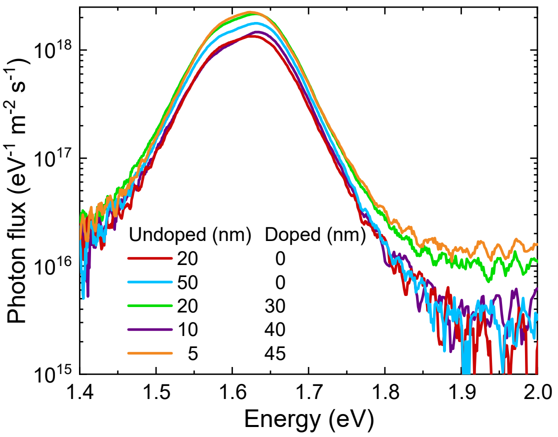

Figure S3. Photoluminescence photon flux of glass | $Cs_{0.05}(FA_{0.75}MA_{0.25})_{0.95}Pb(I_{0.83}Br_{0.17})_3$ | C_{60} layers for different undoped/doped C_{60} bilayers determined for illumination from the glass side. The spectra were used to determine the QFLS shown in Table 1 of the main text.
